# Cadaveric addendum to the consensus on ultrasound‐based lymph‐node staging in gynecological cancer

**DOI:** 10.1002/uog.70197

**Published:** 2026-03-24

**Authors:** D. Fischerova, E. Farsi, E. Facchetti, E. Panizzolo, G. Gambino, H. Reina, F. Sousa, M. R. Kadajari, N. Tiszlavicz, G. Garganese, O. Nanka, Collaborators, E. Gatti, C. Culcasi

**Affiliations:** ^1^ Department of Gynecology, Obstetrics and Neonatology, First Faculty of Medicine Charles University and General University Hospital Prague Czech Republic; ^2^ Obstetrics and Gynecology, Department of Experimental and Clinical Biomedical Sciences University of Florence, Careggi Hospital Florence Italy; ^3^ Obstetrics and Gynecology Clinic, Department of Women and Children's Health University of Padua Padua Italy; ^4^ Department of Gynecologic Oncology, ARNAS Civico Di Cristina Benfratelli University of Palermo Palermo Italy; ^5^ Department of Gynecological Ultrasound and Prenatal Diagnostics, Women's Hospital University Hospital of Basel Basel Switzerland; ^6^ Serviço de Ginecologia/Obstetrícia, Hospital de Cascais Cascais Portugal; ^7^ Obstetrics and Gynaecology Department University Hospital Waterford Waterford Ireland; ^8^ Department of Obstetrics and Gynecology Kiskunhalas Semmelweis Hospital Kiskunhalas Hungary; ^9^ Unità Operativa di Chirurgia dei Genitali Esterni Femminili, Divisione di Ginecologia Oncologica, Dipartimento Scienze della Salute della Donna, del Bambino e di Sanità Pubblica, Fondazione Policlinico Universitario A. Gemelli IRCCS Rome Italy; ^10^ Gemelli Woman Health Center for Digital and Personalized Medicine, Dipartimento Scienze della Vita e Sanità Pubblica, Università Cattolica del Sacro Cuore Rome Italy; ^11^ Institute of Anatomy, First Faculty of Medicine Charles University Prague Czech Republic

## INTRODUCTION

Lymphatic dissemination is a major route of metastatic spread in gynecological malignancies and has direct implications for prognosis and treatment planning^1^. Accurate preoperative identification and characterization of lymph nodes based on imaging are essential for comprehensive clinical staging, intraoperative mapping and individualized therapeutic strategies.

In February 2025, a Consensus Opinion article introduced a structured methodology for ultrasonographic lymph‐node assessment in patients with gynecological cancer, providing a standardized anatomical classification of lymph nodes along with illustrative schematic diagrams and annotated ultrasound videoclips to support clinical implementation^2^. A key element of this approach is the use of standardized lymph‐node descriptors, as defined by the Vulvar International Tumor Analysis group, which ensures uniformity in the assessment and reporting of lymph‐node morphology and vascular features, and contributes to the development of evidence‐based imaging criteria^3^. Together, these tools will enhance the diagnostic utility of ultrasound for regional and distant lymph‐node staging in gynecological cancers and support reproducible reporting in both clinical and research settings.

However, while the Consensus Opinion established a standardized ultrasound methodology, it did not provide direct cadaveric–anatomical correlation. This addendum addresses that gap by presenting detailed dissections of embalmed cadavers, with adherence to *Terminologia Anatomica* ^4^, illustrating the lymph‐node regions described in the original article^2^.

General anatomical descriptions provided in this addendum are based on established references, including *Terminologia Anatomica* ^4^, Gray's Anatomy^5^, Mirilas and Skandalakis^6^, Lengelé *et al*.^7^ and Plutecki *et al*.^8^.

To contextualize this anatomical work, it is important to note that lymphatic vessels predominantly accompany arterial branches, forming perivascular lymphatic plexuses that follow major visceral and parietal arteries toward regional lymph‐node basins^6^. Although lymphatic vessels may also run parallel to venous structures in certain regions, particularly along major collecting pathways such as tributaries of the great saphenous vein or within the retroperitoneum, and near their terminal drainage, arteries serve as the primary anatomical landmarks for lymphatic routing and nodal distribution^6^.

**Figure 1:**
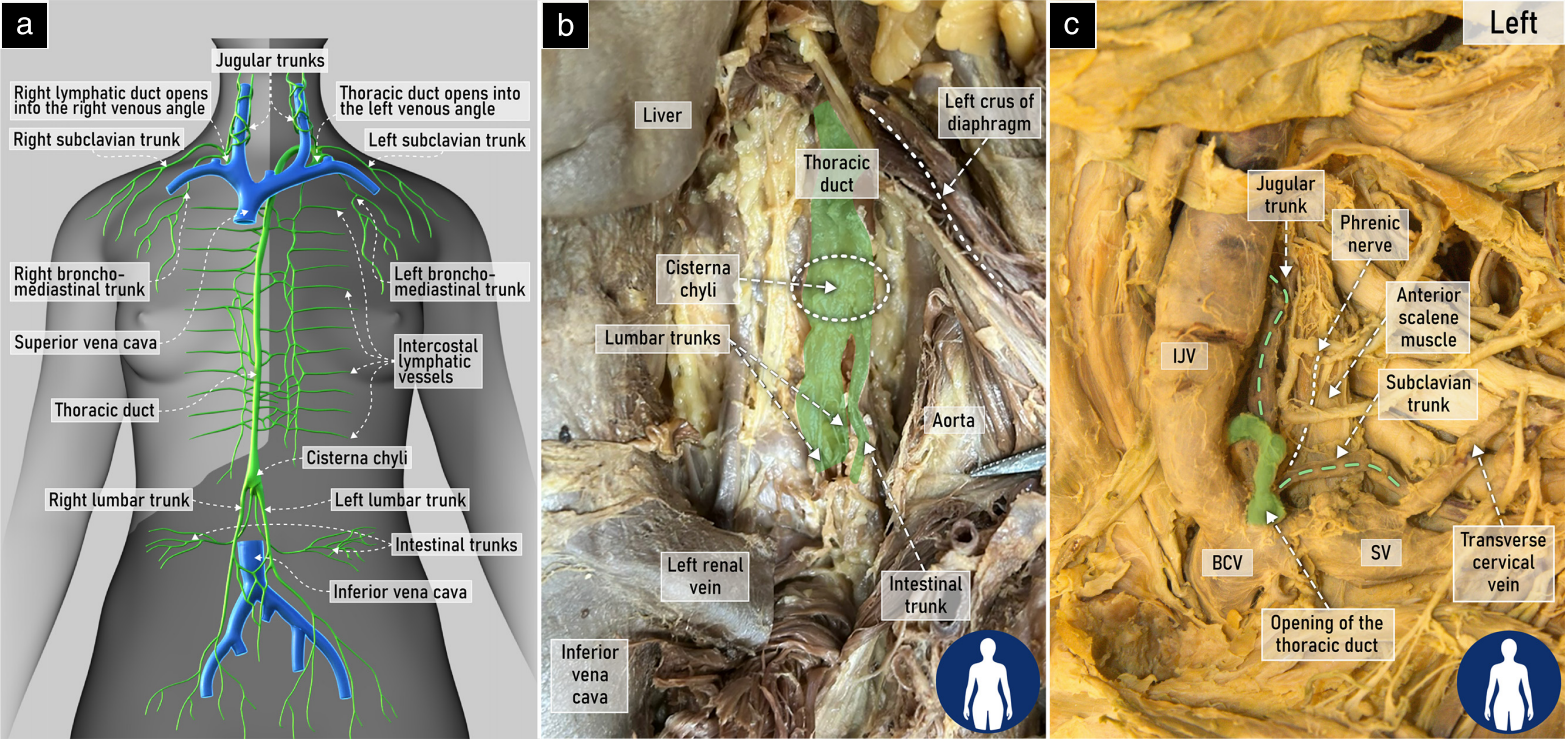
Course of the thoracic duct. (a) Schematic diagram of human lymphatic drainage system (green), showing course of the thoracic duct from its origin at the cisterna chyli to its termination at the left venous angle, which serves as the primary conduit for lymph from the entire lower body, left hemithorax, left upper limb and left side of the head and neck (dark gray). The shorter right lymphatic duct, which drains the right upper quadrant (including right hemithorax, right upper limb and right side of head and neck; light gray) is also indicated^8^. (b) Cadaveric dissection of the abdominal portion of the thoracic duct. The paired lumbar trunks and unpaired intestinal trunk converge to form the cisterna chyli, a dilated sac located adjacent to the right posterolateral aspect of the aorta (retracted laterally in this image). From the cisterna chyli, the thoracic duct (green shading) ascends through the aortic hiatus into the thoracic cavity^8^. (c) Cadaveric dissection of cervical portion of thoracic duct, showing duct coursing posterior to left internal jugular vein (IJV), forming a characteristic arch before termination at the left venous angle. Proximal to its termination, the thoracic duct receives left jugular, left subclavian and left bronchomediastinal trunks (the latter is not visible in this image). BCV, brachiocephalic vein; SV, subclavian vein.

After traversing one or more tiers of lymph nodes, these collecting lymphatic vessels coalesce into larger‐caliber efferent lymphatics, which eventually merge to form the principal lymphatic trunks. In the abdomen, this includes the paired lumbar trunks, which drain the lower limbs, pelvic organs and posterior abdominal wall, and the unpaired intestinal trunk, which drains lymph from the gastrointestinal tract and associated viscera^9^. These major trunks converge to form the cisterna chyli, a dilated sac situated anterolateral to the L1–L2 vertebral bodies, which constitutes the origin of the thoracic duct (Figure 1)^8^. The thoracic duct ascends through the posterior mediastinum and serves as the primary conduit for lymph from the entire lower body. Along its course, it also receives input from the left bronchomediastinal trunk (draining the left hemithorax), the left subclavian trunk (draining the left upper limb) and the left jugular trunk (draining the left side of the head and neck). It ultimately terminates at the left venous angle, which is the junction of the left internal jugular and subclavian veins (Figure 1). In contrast, lymph from the right upper quadrant of the body, including the right side of the head and neck, right upper limb and right hemithorax, drains into the right jugular, subclavian and bronchomediastinal trunks, respectively, which typically converge to form the right lymphatic duct. This short duct empties into the right venous angle, which is the junction of the right internal jugular and subclavian veins (Figure 1)^8^.

The aim of this cadaveric addendum was to provide direct correlation between key ultrasound landmarks defined in the Consensus Opinion and topographic anatomy. By reinforcing the anatomical basis of ultrasound lymph‐node staging, this will support consistent interpretation in both clinical and educational settings, and facilitate treatment planning, including surgery, radiotherapy, systemic therapy and follow‐up imaging.

## METHODOLOGY

The study was performed on eight formaldehyde‐embalmed female cadavers (aged 60–80 years; White ethnicity) at Charles University, Prague, Czech Republic. Bilateral dissections of the relevant lymph‐node regions were carried out in a standardized manner. The main limitation of this approach is the potential influence of embalming artifacts on tissue characteristics.

### Anatomical regions and corresponding lymph‐node basins

The main anatomical regions of interest illustrated in the cadaveric dissection videos (Videoclips [Link], [Link], [Link], [Link], [Link]) and described in the accompanying text provided in Appendix S1 are as follows:

#### Subperitoneal pelvic, retroperitoneal and intraperitoneal spaces and parietal and visceral lymph nodes

Videoclip S1 of cadaveric dissection illustrates the subperitoneal pelvic and retroperitoneal spaces, located caudal and dorsal to the parietal peritoneum, delineating the course of the abdominal aorta, inferior vena cava and their main parietal branches. In addition, the anterior unpaired visceral branches of the abdominal aorta (the celiac trunk (namely the common hepatic and left gastric arteries) and the superior and inferior mesenteric arteries) are shown coursing within the intraperitoneal space before continuing retroperitoneally toward the preaortic (retroperitoneal) space^5^.

The traditional anatomical classification distinguishes abdominopelvic parietal and visceral nodes based on their vascular associations. Parietal lymph nodes are associated with parietal blood vessels. Those located beneath the peritoneum, alongside the aorta, inferior vena cava and their parietal branches (including the iliac vessels), are commonly referred to as abdominal (lumbar) or pelvic (iliac) parietal lymph nodes^4^. In contrast, visceral lymph nodes follow the course of the visceral vessels that supply and drain intra‐abdominal and pelvic viscera, including the urogenital organs^7^. In clinical oncology, both groups of lymph nodes may be sequentially or simultaneously involved in the lymphatic dissemination of gynecological malignancies^2^.

**Figure 2:**
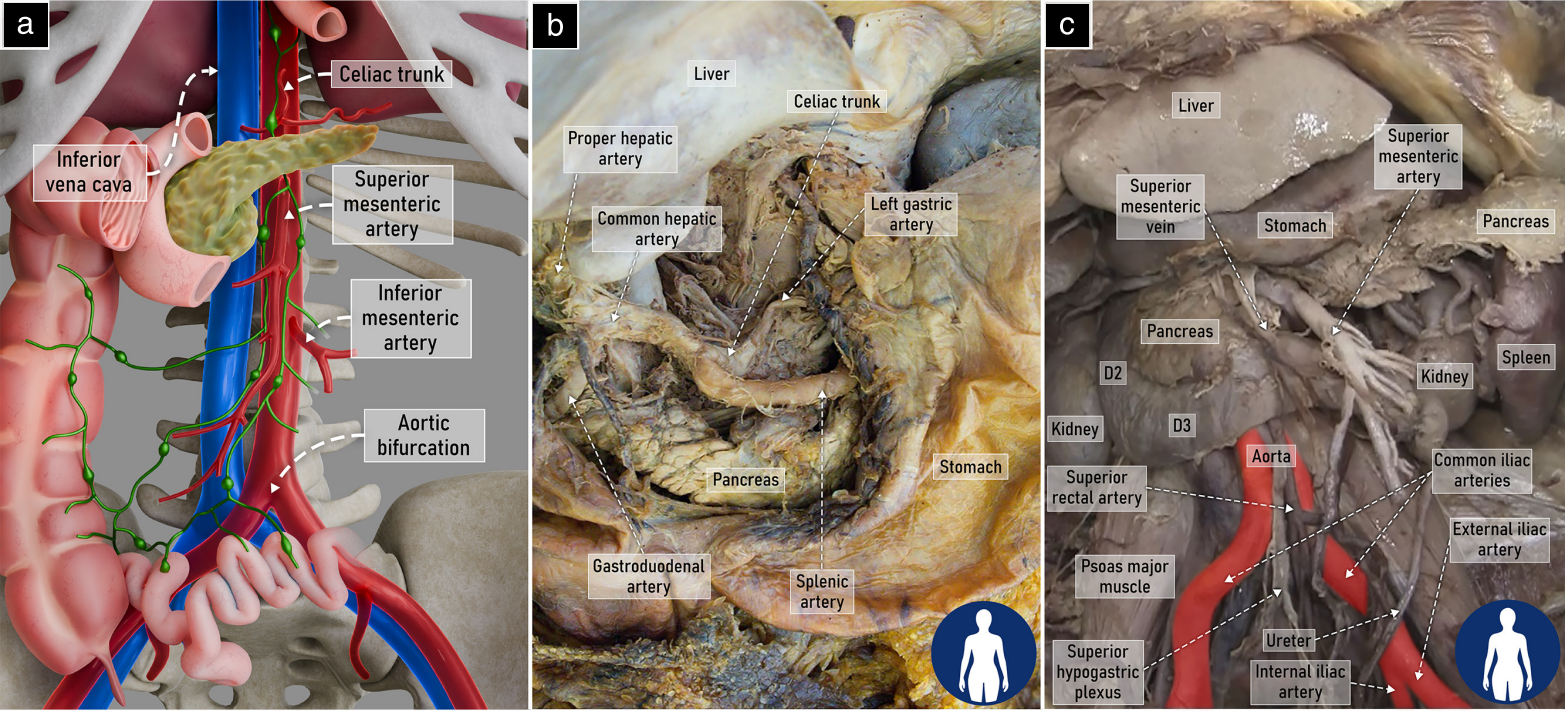
Subperitoneal pelvic, retroperitoneal and intraperitoneal spaces and associated parietal and visceral lymph nodes. (a) Schematic diagram illustrating major retroperitoneal anatomical structures, including the abdominal aorta, inferior vena cava and the origins of the main visceral branches of the anterior aortic wall (celiac trunk, superior mesenteric artery and inferior mesenteric artery). Lymphatic drainage via visceral lymph nodes is depicted from the small intestine (intraperitoneal organ) and colon (retroperitoneal organ). (b) Cadaveric dissection image displaying celiac trunk (first visceral branch of abdominal aorta) and its three main branches: left gastric, splenic and common hepatic arteries. The common hepatic artery further gives rise to gastroduodenal and proper hepatic arteries^5^. These vessels are visualized in an anatomical context with the stomach, pancreas and liver. (c) Cadaveric dissection image showing aortic bifurcation into common iliac arteries, with their subsequent division into external and internal iliac arteries on the left side. External iliac vessels are located adjacent to psoas major muscle. These parietal branches of the abdominal aorta, together with the inferior vena cava (right of the aorta) and its parietal tributaries, serve as landmarks for parietal lymph nodes^5^. Superior mesenteric artery (second visceral branch of abdominal aorta) and superior mesenteric vein are also visible, showing their relationships to the stomach, pancreas and second and third portions of the duodenum (D2 and D3, respectively). The inferior mesenteric artery (third visceral branch of abdominal aorta) arises from the anterior surface of the abdominal aorta approximately 3–4 cm above aortic bifurcation; in this dissection, its terminal branch (superior rectal artery) is demonstrated^5^. Visceral arterial branches, accompanied by venous tributaries to the portal vein (not shown), indicate course of visceral lymphatic drainage pathways. Videoclip S1 shows cadaveric dissection.

In the context of gynecological cancer, the key visceral branches of the internal iliac arteries involved in pelvic lymphatic drainage are the uterine, vaginal, superior vesical, inferior vesical and middle rectal arteries, with involvement of the pelvic visceral lymph nodes^2^, ^10^. In more advanced diseases, lymphatic dissemination may also occur along visceral branches of the abdominal aorta, most notably the celiac trunk, superior mesenteric artery and inferior mesenteric artery, affecting abdominal visceral lymph nodes (Figure 2).

Venous drainage largely mirrors arterial anatomy. Pelvic visceral organs, such as the uterus, vagina and bladder, typically drain directly into the systemic venous circulation via tributaries of the inferior vena cava. In contrast, abdominal unpaired visceral organs, including most of the gastrointestinal tract, drain first into the portal venous system, which carries blood to the liver before it enters the systemic circulation via the hepatic veins^5^.

Lymphatic drainage parallels this organization. Lymph from pelvic visceral organs drains into the pelvic and lower or upper abdominal parietal lymph nodes, with efferent vessels converging into the lumbar trunks. In contrast, afferent lymphatic vessels from unpaired abdominal visceral organs follow the visceral blood vessels, coursing intraperitoneally to reach visceral lymph nodes. Their efferent vessels converge in the preaortic space to form the intestinal lymphatic trunk (Figure 1)^7^, ^11^.

In Videoclip S1, the subperitoneal pelvic and retroperitoneal spaces are first demonstrated, showing the abdominal aorta, inferior vena cava and their major parietal branches, which serve as landmarks for adjacent parietal lymph nodes. The visualization continues caudally into the vascular space beneath the inguinal ligament, where the external iliac artery becomes the femoral artery on both sides, and the femoral veins drain into the external iliac veins^5^. Finally, the continuity between the retroperitoneal and intraperitoneal spaces is demonstrated through the course of the anterior unpaired visceral branches of the abdominal aorta. The course of the visceral branches of the abdominal aorta accompanied by venous tributaries to the portal vein indicates the location of the corresponding visceral lymph nodes.

#### Femoral triangle and inguinal lymph nodes

**Figure 3:**
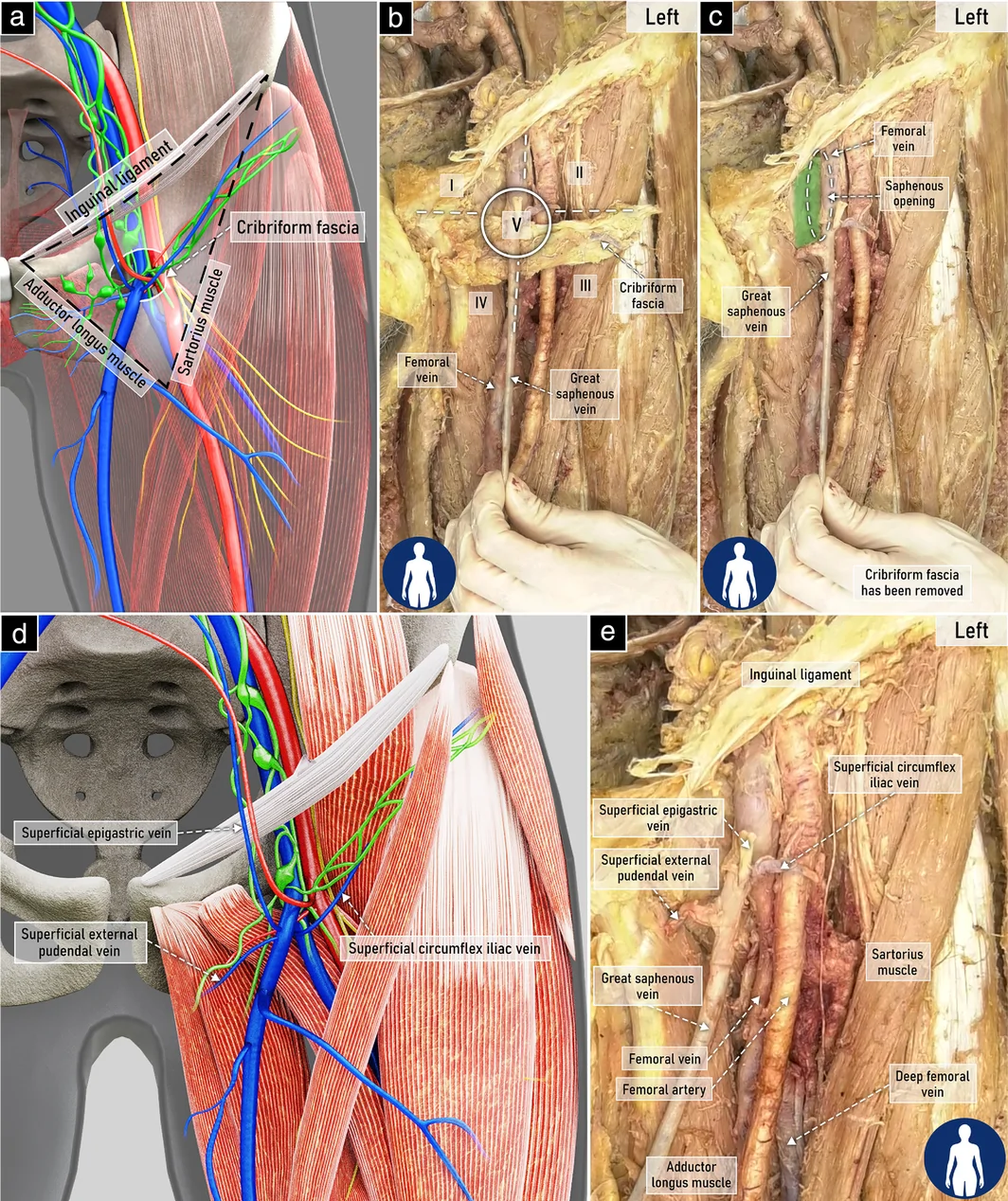
Femoral triangle and associated inguinal lymph nodes. (a) Schematic diagram of left femoral triangle showing anatomical location of inguinal lymph nodes (green) along tributaries of the great saphenous vein and femoral vein. Superficial inguinal lymph nodes are situated superficial to cribriform fascia, while deep lymph nodes lie beneath it^2^. (b) Cadaveric dissection image showing the five Daseler regions, defined by virtual vertical line along the femoral vein and perpendicular horizontal line at the level of the saphenofemoral junction. Circle around intersection defines central region (V). Other four regions are: superomedial (I); superolateral (II); inferolateral (III); and inferomedial (IV)^2^, ^13^. (c) Dissection image after removal of cribriform fascia, demonstrating location of deep inguinal lymph nodes (green shading). (d) Schematic diagram showing tributaries of great saphenous vein: superficial external pudendal vein, superficial circumflex iliac vein and superficial epigastric vein (left side). (e) Cadaveric dissection image showing same venous tributaries as (d) *in situ*, with surrounding anatomical landmarks of sartorius muscle, adductor longus muscle and inguinal ligament, which define the boundaries of the femoral triangle^2^, ^5^. Femoral artery, femoral vein and great saphenous vein are also shown. Videoclip S2 shows cadaveric dissection.

Videoclip S2 of cadaveric dissection demonstrates the left femoral triangle, also known as Scarpa's triangle (Figure 3). The base of the triangle is formed by the inguinal ligament; the lateral border is defined by the medial edge of the sartorius muscle and its medial border by the lateral edge of the adductor longus muscle. The floor is formed by the iliopsoas muscle laterally and the pectineus muscle medially, with the apex located caudally at the intersection of the sartorius and adductor longus muscles^2^.

The lymph nodes within the femoral triangle are classified into superficial and deep inguinal lymph‐node groups, separated by the cribriform fascia^2^, ^3^. The superficial inguinal lymph nodes are situated in the space between the superficial fascia (Camper's fascia) and the deep fascia (fascia lata) and lie along the great saphenous vein and its tributaries: the superficial external pudendal vein, superficial circumflex iliac vein and superficial epigastric vein^2^. The deep inguinal lymph nodes lie exclusively within the fossa ovalis, beneath the cribriform fascia, cranial to the saphenofemoral junction and medial to the femoral vein. The uppermost node of this group is the node of Cloquet (Rosenmüller's node), which is located at the upper end of the femoral canal and represents the anatomical transition between the deep inguinal and external iliac lymph‐node groups^12^. The femoral triangle can be subdivided into five Daseler regions (Figure 3b)^13^.

Videoclip S2 provides a detailed demonstration of the anatomical boundaries of the femoral triangle, with particular emphasis on the main vascular structures and their tributaries, along which the lymph nodes are distributed.

#### Anterior, middle and posterior mediastinum, and superior diaphragmatic (cardiophrenic) lymph nodes

**Figure 4:**
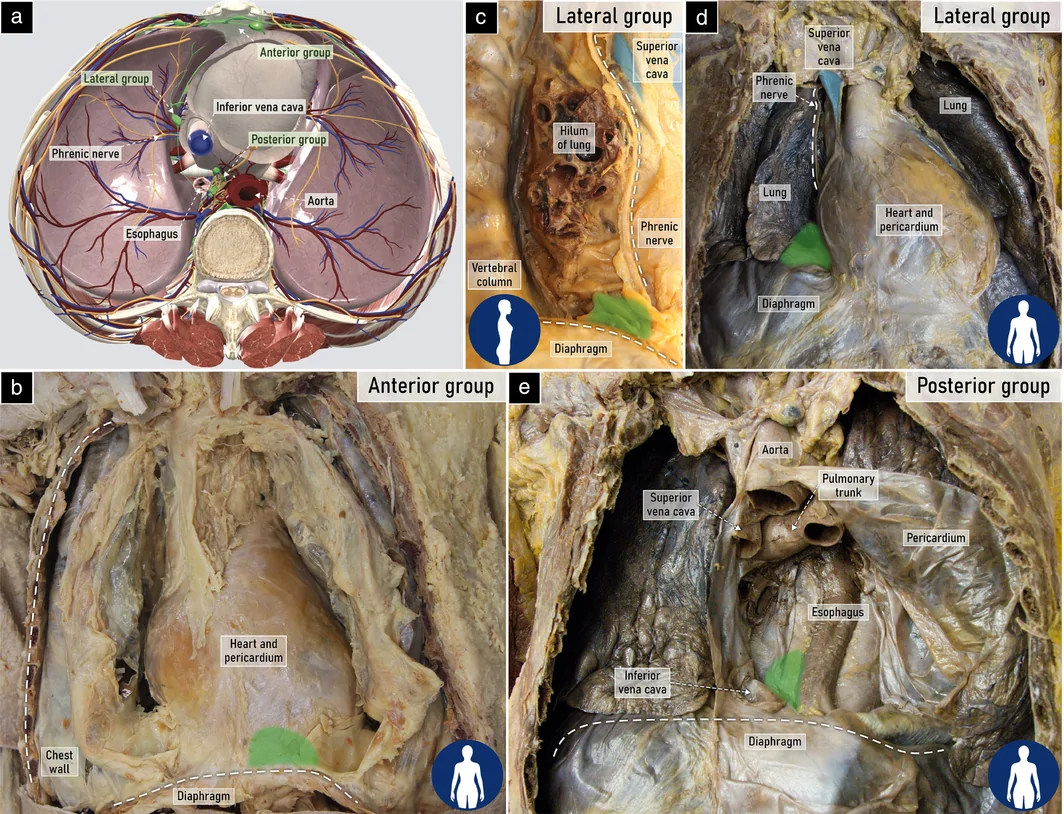
Anterior, middle and posterior mediastinum and associated superior diaphragmatic (cardiophrenic) lymph nodes. (a) Schematic illustration in inferior view showing anatomical location of superior diaphragmatic (cardiophrenic) lymph nodes, categorized into anterior, lateral and posterior groups. (b) Cadaveric dissection in anterior view showing anterior lymph node group (green shading) situated ventral to the pericardium and the base of the heart (right chest wall and diaphragm demarcated by dashed line)^5^. (c,d) Cadaveric dissections in sagittal (c) and anterior (d) views showing lateral lymph‐node group (green shading), located adjacent to the phrenic nerve and near the hilum of the lung (right phrenic nerve and diaphragm indicated with dashed lines)^5^. In these sections, only the right lateral group is visible. (e) Cadaveric dissection in anterior view showing posterior lymph‐node group (green shading) positioned near the inferior vena cava and esophagus (diaphragm indicated with dashed line)^5^. To improve visibility, anterior chest wall, including sternum, has been removed. Videoclip S3 shows cadaveric dissection.

Videoclip S3 of cadaveric dissection demonstrates the anterior, middle and posterior compartments of the inferior mediastinum, where the superior diaphragmatic (cardiophrenic) lymph nodes are located within the fatty tissue above the diaphragm, in the lowermost part of the mediastinum. This region is bordered inferiorly by the diaphragm, anteriorly by the chest wall, medially by the pericardium, laterally by the mediastinal pleura and phrenic nerves, dorsally by the esophagus and adjacent mediastinal structures and superiorly by the inferior pericardial reflections (Figure 4)^5^.

The superior diaphragmatic lymph nodes can be categorized into three groups based on their location and lymphatic drainage territory: anterior, lateral and posterior (Figure 4). The anterior group is situated between the xiphoid process of the sternum and the costochondral articulation of the seventh rib, and the anterior surface of the pericardium. The lateral group lies between the pericardium medially and the lung hilum laterally, adjacent to the course of the phrenic nerve. The posterior group is positioned adjacent to the esophagus, posteromedial to the inferior vena cava^5^.

The cadaveric dissection shown in Videoclip S3 highlights the location of these nodal groups and their spatial relationships with adjacent anatomical structures.

#### Supraclavicular fossa and supraclavicular lymph nodes

**Figure 5:**
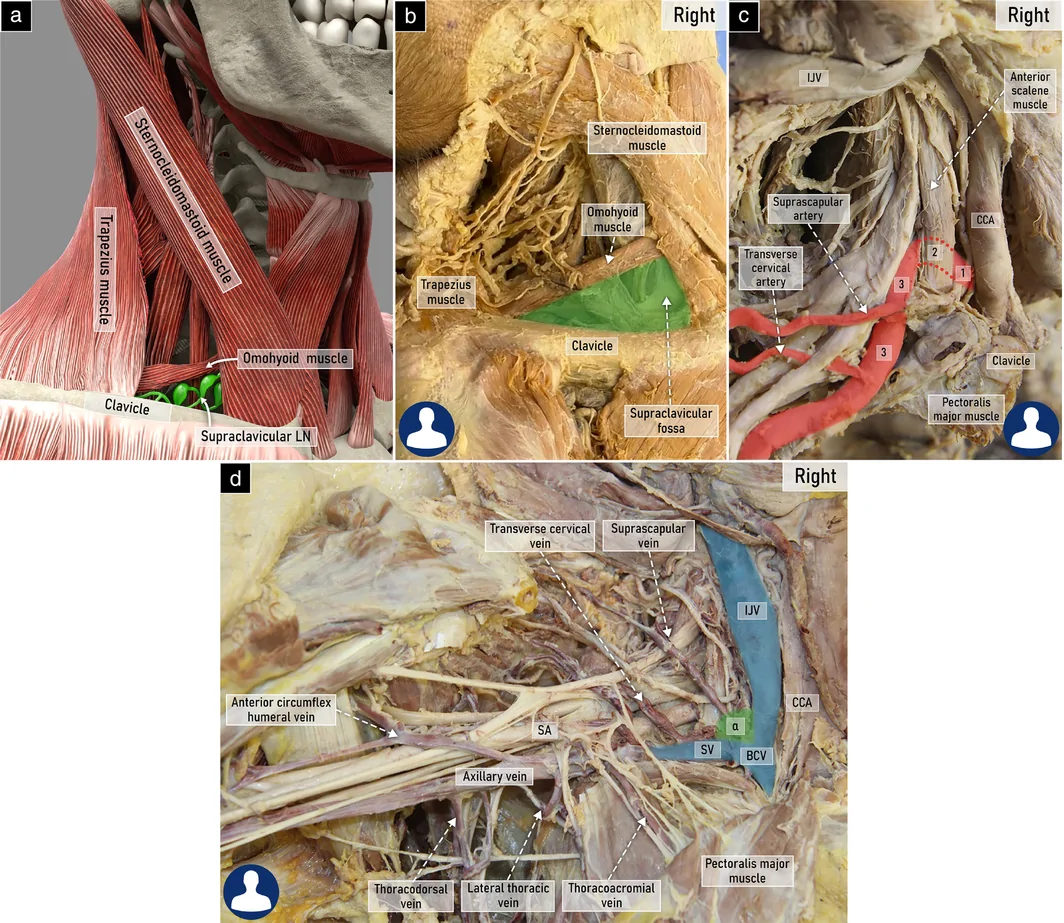
Supraclavicular fossa and associated supraclavicular lymph nodes (LN). (a) Schematic diagram demonstrating anatomical location of right supraclavicular LN (green) within supraclavicular fossa, bordered cranially by inferior belly of omohyoid muscle, caudally by superior border of clavicle, medially by posterior border of sternocleidomastoid muscle and laterally by anterior border of trapezius muscle^5^. (b) Cadaveric dissection image (right side) showing topographic boundaries of supraclavicular fossa (green shading), using the same anatomical landmarks as in (a). (c) Cadaveric dissection image showing anatomical divisions of the subclavian artery relative to anterior scalene muscle: interscalenic (1) (medial to muscle), intrascalenic (2) (posterior to muscle) and extrascalenic (3) (lateral to muscle). Also visible are key branches of the thyrocervical trunk, including transverse cervical and suprascapular arteries, which course anterior to brachial plexus roots. The internal jugular vein (IJV) is retracted superiorly to expose underlying anatomical structures. (d) Detailed cadaveric dissection image of right venous angle (α), (highlighted by green shading), where IJV joins the subclavian vein (SV) to form the brachiocephalic vein (BCV) (these venous structures are highlighted by blue shading). Suprascapular and transverse cervical veins draining into SV can also be seen. Additionally, the axillary vein and several of its tributaries are visible, including thoracoacromial, lateral thoracic, thoracodorsal and anterior circumflex humeral veins^5^. The spatial relationship of the right venous angle to the subclavian artery (SA) and common carotid artery (CCA) is also depicted. Videoclip S4 shows cadaveric dissection.

Videoclip S4 of cadaveric dissection demonstrates the supraclavicular fossa, delimited cranially by the omohyoid muscle, caudally by the superior border of the clavicle, laterally by the anterior border of the trapezius muscle and medially by the posterior border of the sternocleidomastoid muscle (Figure 5)^5^.

The supraclavicular lymph nodes, which are part of the lateral cervical region lymph‐node group, reside within the supraclavicular fossa, posterolateral to the internal jugular vein. While present bilaterally, these nodes are more frequently infiltrated on the left side owing to the anatomical course and termination of the thoracic duct into the left venous angle (Figure 1)^2^. In gynecological cancers, as well as in other malignancies (such as gastric, pancreatic, lung, breast, testicular and renal cancers), enlargement of the supraclavicular node, also known as Virchow's node, which is located adjacent to the opening of the thoracic duct into the venous angle, may represent the first clinical sign of occult metastatic spread, a phenomenon referred to as Troisier's sign^5^. The right lymphatic duct terminates analogously at the right venous angle (Figure 5).

The subclavian artery is an important anatomical structure in this region. It courses posteriorly to the anterior scalene muscle and is classically divided into three parts relative to this muscle: medial (interscalenic), posterior (intrascalenic) and lateral (extrascalenic) (Figure 5). The medial part gives rise to the thyrocervical trunk, which lies close to the arch of the thoracic duct on the left (Figure 1) and the terminal portion of the right lymphatic duct on the right^5^.

In Videoclip S4 the boundaries and contents of the supraclavicular fossa, also referred to as the omoclavicular triangle, are illustrated in detail.

#### Axilla and anterior thoracic wall, and axillary and parasternal lymph nodes

**Figure 6:**
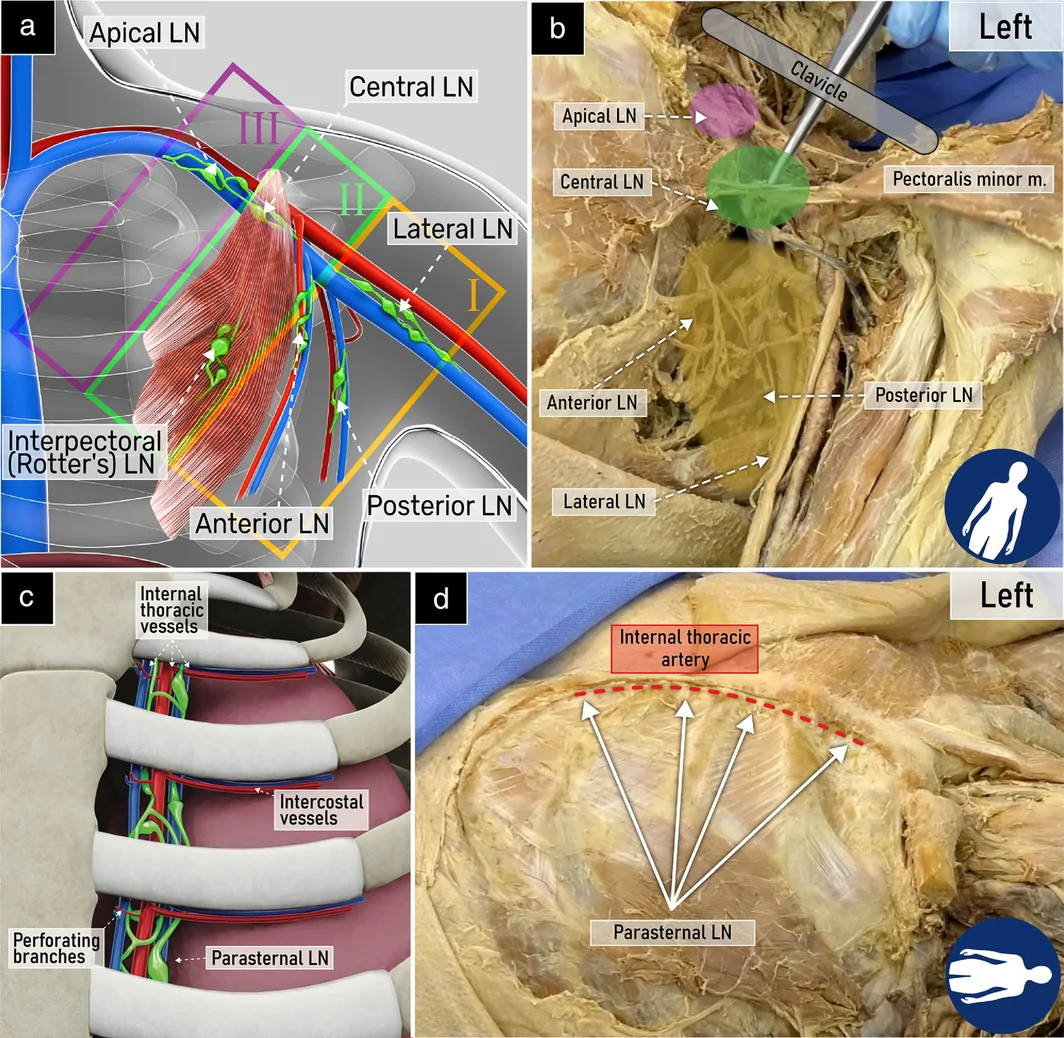
Axilla and anterior thoracic wall and associated axillary and parasternal lymph nodes (LN). (a) Schematic diagram illustrating anatomical classification of axillary LN (green) into three levels based on their relationship to pectoralis minor muscle: Level I (lateral, anterior and posterior LN groups), Level II (central LN group) and Level III (apical LN group). Interpectoral (Rotter's nodes) LN group (which is not part of Level II) is also shown^2^. (b) Cadaveric dissection image (left side) depicting three axillary LN levels. Clavicle has been removed for better visualization, with its anatomical position indicated by gray overlay. The pectoralis minor muscle (m) is retracted superiorly to expose the underlying central LN group (green shading). Lateral, anterior and posterior LN groups (yellow shading) and apical LN group (purple shading) are also indicated. (c) Schematic diagram of parasternal (internal mammary) LN (green), located within anterior thoracic wall, alongside internal thoracic vessels. (d) Cadaveric dissection image of left anterior thoracic wall showing projected course of internal thoracic artery (red dashed line), along which parasternal LN are located. Internal thoracic artery and LN are not directly seen in image. Videoclip S5 shows cadaveric dissection.

Videoclip S5 of cadaveric dissection presents the axilla, a truncated pyramidal anatomical space located between the thoracic wall and the upper limb. Its anatomical boundaries, comprising muscular, osseous and fascial structures, are shown in detail in Figure 6.

The axillary artery transitions from the subclavian artery at the first rib and then becomes the brachial artery at the humeral insertion of the teres major muscle. Axillary lymph nodes are located along the course of the axillary vessels and are classified into three levels based on their anatomical relationship with the pectoralis minor muscle. Level‐I lymph nodes are situated lateral to the pectoralis minor muscle and include the lateral (humeral), posterior (subscapular) and anterior (pectoral) lymph nodes. Level‐II lymph nodes lie deep to the pectoralis minor muscle, between its medial and lateral borders, and include the central lymph nodes located along the middle part of the axillary artery. Intrapectoral lymph nodes (Rotter's nodes), situated between the pectoralis major and minor muscles, do not belong to axillary Level II but represent a distinct nodal group relevant in the dissemination of breast cancer. Level‐III lymph nodes are positioned medial to the pectoralis minor muscle, near the apex of the axilla^2^, ^5^.

In Videoclip S5, the boundaries and contents of the axilla are illustrated in detail, along with the anterior thoracic wall and intercostal spaces. The parasternal lymph nodes are also shown in schematic form, located within the anterior thoracic wall, alongside the internal thoracic (mammary) artery (Figure 6c).

## DISCUSSION

**Figure 7:**
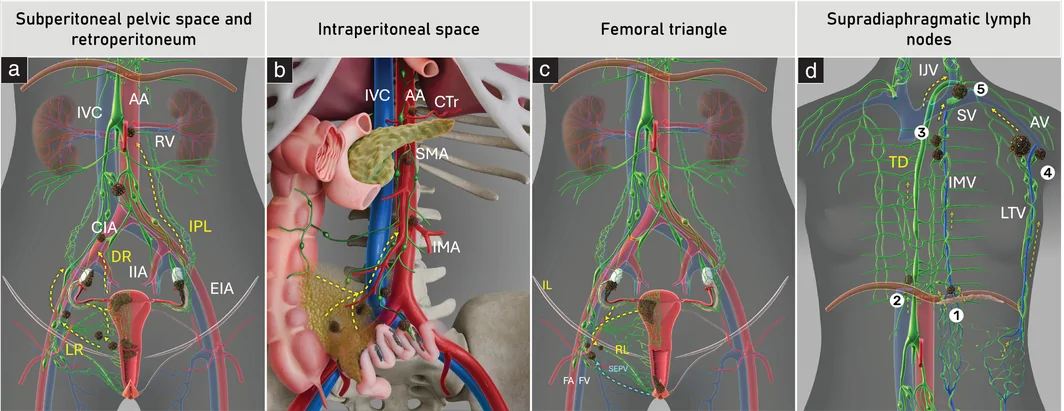
Schematic diagram of key lymph‐node basins in gynecological cancers, illustrating vascular landmarks and patterns of spread. (a) Subperitoneal pelvic space and retroperitoneum: pelvic (iliac) and abdominal (lumbar) lymph nodes involved in gynecological cancers. Main lymphatic routes are indicated (dashed yellow lines). Two pelvic lymphatic routes are shown: lateral route (LR) along uterine vessels mainly to external iliac lymph nodes and dorsal route (DR) draining to internal iliac lymph nodes and presacral area^14^. Abdominal lymph nodes may be affected either through secondary spread from iliac lymph nodes or directly via lymphatic vessels along the infundibulopelvic ligament (IPL) up to level of renal veins (RV)^15^. (b) Intraperitoneal space: lymphatic drainage from unpaired abdominal viscera follows visceral branches of abdominal aorta (AA) to preaortic lymph nodes, typically involved in advanced disease with visceral (intestinal) carcinomatosis. The figure illustrates visceral carcinomatosis on the serosal surface of the small bowel, together with infiltrated lymph nodes distributed along the visceral branches of the AA. The direction of lymphatic flow is indicated by the dashed yellow line^16^, ^17^. (c) Femoral triangle: inguinal lymph nodes may be involved through distinct pathways via lymphatics accompanying round ligament (RL) (yellow dashed line), superficial external pudendal vessels (SEPV) (blue dashed line) or anterior parietal pathway^18^, ^19^, ^27^. The RL pathway is relevant in advanced tumors involving the upper uterus (endometrial or tubo‐ovarian cancers), the SEPV pathway is characteristic of vulvar and lower‐vaginal cancers and the anterior pathway is mainly involved in advanced ovarian cancer with parietal carcinomatosis^27^. (d) Supradiaphragmatic lymph‐node basins: anterior (1) and posterior (2) superior diaphragmatic (cardiophrenic), parasternal (3), axillary (4) and supraclavicular (5) lymph nodes may be affected through either the posterior (retroperitoneal) or the anterior (parietal) lymphatic pathway. The posterior pathway includes drainage along the thoracic duct (TD) to posterior superior diaphragmatic (2) and supraclavicular (5) lymph nodes on the left side. The anterior pathway includes lymphatic drainage along the internal mammary vessels to anterior superior diaphragmatic (cardiophrenic) lymph nodes (1), which are often also involved via transdiaphragmatic spread, and to parasternal lymph nodes (3), as well as along the lateral thoracic vessels to axillary lymph nodes (4), which, via their respective lymphatic trunks (Figure 1), converge at the ipsilateral jugulosubclavian venous angle^7^, ^20^, ^27^. AV, axillary vein; CIA, common iliac artery; CTr, celiac trunk; EIA, external iliac artery; FA, femoral artery; FV, femoral vein; IIA, internal iliac artery; IJV, internal jugular vein; IL, inguinal ligament; IMA, inferior mesenteric artery; IMV, internal mammary vein; IVC, inferior vena cava; LTV, lateral thoracic vein; SMA, superior mesenteric artery; SV, subclavian vein.

This cadaveric addendum may have implications for future education, the development of guidelines and technological innovation. By providing direct anatomical correlation to standardized ultrasonographic methodology, it will serve as a valuable teaching resource that can be integrated into structured training modules for gynecological oncologists, radiologists and sonographers. Such anatomical–imaging correlations could be incorporated into future updates of international guidelines to reinforce consistent lymph‐node assessment across centers. Furthermore, the anatomically accurate reference maps generated in this work may serve as a foundation for the development of three‐dimensional ultrasound training simulators and for training datasets in artificial intelligence applications, for which accurate anatomical annotation is essential for automated lymph‐node detection and classification using imaging. Table 1 and Figure 7 summarize the principal nodal basins relevant to gynecological oncology, with their vascular landmarks and associated malignancies categorized into locoregional and distant spread.

**Table 1:** Summary of nodal basins with vascular landmarks and associated gynecological malignancies

Anatomical region	Nodal basins	Key vascular landmarks	Locoregional spread	Distant spread
*Infradiaphragmatic lymph nodes*				
Subperitoneal pelvic space	Pelvic (iliac) parietal and visceral lymph nodes*	1. Internal iliac vessels 2. External iliac vessels 3. Common iliac vessels	Ovarian^21^, cervical^22^, endometrial^23^ and vaginal^24^ cancers	Vulvar cancer^25^
Retroperitoneal space	Abdominal (lumbar) lymph nodes†	1. Abdominal aorta 2. Inferior vena cava	Ovarian^21^, cervical^22^ and endometrial^23^ cancers	Vaginal^24^ and vulvar^25^ cancers
Intraperitoneal space	Abdominal (visceral) lymph nodes‡	1. Anterior unpaired visceral branches of the abdominal aorta: celiac trunk (namely, common hepatic and left gastric arteries) and superior and inferior mesenteric arteries	Ovarian cancer^26^	Cervical^22^, endometrial^23^, vaginal^24^ and vulvar^25^ cancers
Femoral triangle	Inguinal superficial and deep lymph nodes§	1. Great saphenous vein and tributaries 2. Femoral vessels	Vulvar^25^ and vaginal^24^ cancers	Ovarian^21^, cervical^22^ and endometrial^23^ cancers
*Supradiaphragmatic lymph nodes*				
Inferior mediastinum	Superior diaphragmatic (cardiophrenic) lymph nodes¶	1. Internal thoracic (mammary) vessels (anterior group) 2. Inferior vena cava (posterior group)	—	All gynecological cancers^21^, ^22^, ^23^, ^24^, ^25^
Axilla and anterior thoracic wall	Axillary and parasternal lymph nodes††	1. Axillary vessels and their branches 2. Internal thoracic (mammary) vessels	—	All gynecological cancers^21^, ^22^, ^23^, ^24^, ^25^
Supraclavicular fossa	Supraclavicular lymph nodes**	1. Internal jugular vein 2. Subclavian vessels	—	All gynecological cancers^21^, ^22^, ^23^, ^24^, ^25^

* Internal iliac lymph nodes are located along visceral and parietal branches of internal iliac vessels; external iliac lymph nodes are located along external iliac artery and vein, and common iliac lymph nodes are located along common iliac vessels up to aortic bifurcation^5^.

† Abdominal (lumbar) lymph nodes are grouped as left lumbar (lateral aortic, preaortic and retroaortic), right lumbar (lateral caval, precaval and retrocaval) and intermediate lumbar groups^4^. Preaortic lymph nodes, situated on anterior aspect of abdominal aorta adjacent to origin of celiac trunk and superior and inferior mesenteric arteries, drain predominantly via intestinal trunk; others drain via lumbar trunks^7^.

‡ Abdominal visceral lymph nodes (in intraperitoneal space) accompany peripheral anterior unpaired visceral branches of abdominal aorta (celiac trunk (namely the common hepatic and left gastric arteries) and superior and inferior mesenteric arteries), thus are intraperitoneal until their ascent to root of vessels on anterior aspect of abdominal aorta, where they become retroperitoneal^5^.

§ Inguinal lymph nodes (in femoral triangle) include superficial lymph nodes along course of great saphenous vein and its tributaries and deep lymph nodes medial to femoral vein within fossa ovalis (including uppermost node of Cloquet at femoral canal)^4^, ^12^.

¶ Superior diaphragmatic (cardiophrenic) lymph nodes (in inferior mediastinum) are divided into three groups: anterior lymph nodes along the internal thoracic (mammary) vessels, posterior lymph nodes adjacent to the inferior vena cava, esophagus or descending aorta and lateral lymph nodes without a clear vascular landmark on ultrasound^2^. The lateral group does not lie on the principal gynecological lymphatic pathways and is only exceptionally involved in gynecological malignancies.

** Supraclavicular lymph nodes (in supraclavicular fossa) are adjacent to the posterolateral aspect of the internal jugular vein at the venous angle, adjacent to the subclavian vessels^2^.

†† Axillary lymph nodes are organized around axillary vessels and their branches (lateral thoracic, subscapular and thoracodorsal)^2^. Parasternal lymph nodes accompany internal thoracic (mammary) vessels along sternum^5^.

### Conclusions

This cadaver‐based anatomical supplement provides a clear visual guide to lymph‐node regions relevant to gynecological oncology, complementing the ultrasound methodology introduced in the recent Consensus Opinion. By linking sonographic landmarks with anatomical analogs from dissections, it will enhance the understanding of lymph‐node basins and their organization. Beyond its educational value for trainees and specialists in gynecological oncology and imaging, precise anatomical knowledge also strengthens multidisciplinary collaboration, supporting accurate staging and informed therapeutic decisions.

## Supporting information


**Appendix S1** Description of videoclips.

**Videoclip S1** Cadaveric dissection showing subperitoneal pelvic, retroperitoneal and intraperitoneal spaces and parietal and visceral lymph nodes.

**Videoclip S2** Cadaveric dissection showing femoral triangle and inguinal lymph nodes.

**Videoclip S3** Cadaveric dissection showing anterior, middle and posterior mediastinum and superior diaphragmatic (cardiophrenic) lymph nodes.

**Videoclip S4** Cadaveric dissection showing supraclavicular fossa and supraclavicular lymph nodes.

**Videoclip S5** Cadaveric dissection showing axilla and anterior thoracic wall and axillary and parasternal lymph nodes.

## Data Availability

Data sharing not applicable to this article as no datasets were generated or analysed during the current study.

## References

[1] Pano B , Sebastia C , Ripoll E , et al. Pathways of lymphatic spread in gynecologic malignancies. Radiographics. 2015;35(3):916‐945. 25969940 10.1148/rg.2015140086

[2] Fischerova D , Gatti E , Culcasi C , et al. Ultrasound assessment of lymph nodes for staging of gynecological cancer: consensus opinion on terminology and examination technique. Ultrasound Obstet Gynecol. 2025;65(2):206‐225. 39513930 10.1002/uog.29127 PMC12133214

[3] Fischerova D , Garganese G , Reina H , et al. Terms, definitions and measurements to describe sonographic features of lymph nodes: consensus opinion from the Vulvar International Tumor Analysis (VITA) group. Ultrasound Obstet Gynecol. 2021;57(6):861‐879. 34077608 10.1002/uog.23617

[4] FIPAT , ed. Terminologia Anatomica. Vol 2019. 2nd ed. *FIPAT.library.dal.ca. Federative International Programme for Anatomical Terminology* ; 2019.

[5] Standring S , Gray H . Gray's anatomy: the anatomical basis of clinical practice. Vol xvii. 42nd ed. Elsevier; 2021:1588.

[6] Mirilas P , Skandalakis JE . Surgical anatomy of the retroperitoneal spaces, Part III: Retroperitoneal blood vessels and lymphatics. Am Surg. 2010;76(2):139‐144. 20336888

[7] Lengelé B , Nyssen‐Behets C , Scalliet P . Anatomical bases for the radiological delineation of lymph node areas. Upper limbs, chest and abdomen. Radiother Oncol. 2007;84(3):335‐347. 17719668 10.1016/j.radonc.2007.07.016

[8] Plutecki D , Bonczar M , Wilk J , et al. The anatomy of the thoracic duct and cisterna chyli: a meta‐analysis with surgical implications. J Clin Med. 2024;13(15):4285. 39124550 10.3390/jcm13154285 PMC11313251

[9] Lengele B , Hamoir M , Scalliet P , Gregoire V . Anatomical bases for the radiological delineation of lymph node areas. Major collecting trunks, head and neck. Radiother Oncol. 2007;85(1):146‐155. 17383038 10.1016/j.radonc.2007.02.009

[10] Fischerova D , Culcasi C , Gatti E , Ng Z , Burgetova A , Szabo G . Ultrasound assessment of the pelvic sidewall: methodological consensus opinion. Ultrasound Obstet Gynecol. 2025;65(1):94‐105. 39499650 10.1002/uog.29122 PMC11693842

[11] Dvoretsky PM , Richards KA , Angel C , et al. Distribution of disease at autopsy in 100 women with ovarian cancer. Hum Pathol. 1988;19(1):57‐63. 3335391 10.1016/s0046-8177(88)80316-2

[12] Way S . The anatomy of the lymphatic drainage of the vulva and its influence on the radical operation for carcinoma. Ann R Coll Surg Engl. 1948;3(4):187‐209. 18889533 PMC2238289

[13] Daseler EH , Anson BJ , Reimann AF . Radical excision of the inguinal and iliac lymph glands; a study based upon 450 anatomical dissections and upon supportive clinical observations. Surg Gynecol Obstet. 1948;87(6):679‐694. 18120502

[14] Geppert B , Lonnerfors C , Bollino M , Arechvo A , Persson J . A study on uterine lymphatic anatomy for standardization of pelvic sentinel lymph node detection in endometrial cancer. Gynecol Oncol. 2017;145(2):256‐261. 28196672 10.1016/j.ygyno.2017.02.018

[15] Takeshima N , Hirai Y , Umayahara K , Fujiwara K , Takizawa K , Hasumi K . Lymph node metastasis in ovarian cancer: difference between serous and non‐serous primary tumors. Gynecol Oncol. 2005;99(2):427‐431. 16112718 10.1016/j.ygyno.2005.06.051

[16] Gallotta V , Fanfani F , Fagotti A , et al. Mesenteric lymph node involvement in advanced ovarian cancer patients undergoing rectosigmoid resection: prognostic role and clinical considerations. Ann Surg Oncol. 2014;21(7):2369‐2375. 24558070 10.1245/s10434-014-3558-0

[17] O'Hanlan KA , Kargas S , Schreiber M , et al. Ovarian carcinoma metastases to gastrointestinal tract appear to spread like colon carcinoma: implications for surgical resection. Gynecol Oncol. 1995;59(2):200‐206. 7590473 10.1006/gyno.1995.0008

[18] Pavlista D , Eliska O . Superficial lymphatic drainage of the vulva and its relation to the regional nodes: an experimental study. Folia Morphol (Warsz). 2022;81(4):917‐922. 34590298 10.5603/FM.a2021.0096

[19] Odagiri T , Watari H , Kato T , et al. Distribution of lymph node metastasis sites in endometrial cancer undergoing systematic pelvic and para‐aortic lymphadenectomy: a proposal of optimal lymphadenectomy for future clinical trials. Ann Surg Oncol. 2014;21(8):2755‐2761. 24705578 10.1245/s10434-014-3663-0

[20] Agusti N , Bonaldo G , Kahn RM , et al. Cardiophrenic lymph nodes in advanced ovarian cancer. Int J Gynecol Cancer. 2023;34(1):150‐158. 10.1136/ijgc-2023-004963 38097346

[21] Berek JS , Renz M , Kehoe S , Kumar L , Friedlander M . Cancer of the ovary, fallopian tube, and peritoneum: 2021 update. Int J Gynaecol Obstet. 2021;155 Suppl 1(Suppl 1):61‐85. 34669199 10.1002/ijgo.13878 PMC9298325

[22] Bhatla N , Aoki D , Sharma DN , Sankaranarayanan R . Cancer of the cervix uteri: 2021 update. Int J Gynaecol Obstet. 2021;155 Suppl 1(Suppl 1):28‐44. 34669203 10.1002/ijgo.13865 PMC9298213

[23] Berek JS , Matias‐Guiu X , Creutzberg C , et al. FIGO staging of endometrial cancer: 2023. Int J Gynaecol Obstet. 2023;162(2):383‐394. 37337978 10.1002/ijgo.14923

[24] Adams TS , Rogers LJ , Cuello MA . Cancer of the vagina: 2021 update. Int J Gynaecol Obstet. 2021;155 Suppl 1(Suppl 1):19‐27. 10.1002/ijgo.13867 PMC9298013 34669198

[25] Olawaiye AB , Cuello MA , Rogers LJ . Cancer of the vulva: 2021 update. Int J Gynaecol Obstet. 2021;155 Suppl 1(Suppl 1):7‐18. 34669204 10.1002/ijgo.13881 PMC9298362

[26] Prat J . Ovarian, fallopian tube and peritoneal cancer staging: Rationale and explanation of new FIGO staging 2013. Best Pract Res Clin Obstet Gynaecol. 2015;29(6):858‐869. 25890882 10.1016/j.bpobgyn.2015.03.006

[27] Fischerova D , Facchetti E , Panizzolo E , et al. Lymphatic dissemination in gynecologic malignancies : major anatomical pathways. Int J Gynecol Cancer. 2026 (In press). 10.1016/j.ijgc.2026.104645 41980434

