# Supplementary material for: Cadaveric addendum to the consensus on ultrasound‐based lymph‐node staging in gynecological cancer
Source: Ultrasound Obstet Gynecol. 2026 Mar 24;68(3):309–18. doi: 10.1002/uog.70197 (PMC13532176; doi:10.1002/uog.70197)
Supplement: Supplementary file 1 [file UOG-68-309-s004.docx]

**S1 - Retroperitoneum  and subperitoneal pelvic space**

The abdominal aorta originates at the aortic hiatus of the diaphragm at the level of the intervertebral disc between the twelfth thoracic and the first lumbar vertebrae.

It continues caudally, ventral to the lumbar vertebrae before the bifurcation into the 2 common iliac arteries at the level of the 4th lumbar vertebrae.

The right common iliac artery is approximately 1 cm longer than the contralateral because the bifurcation is positioned slightly to the left of the midline.

The external and internal iliac arteries originate from the common iliac artery at the level of the sacroiliac joints. The external iliac artery runs laterally along the medial border of the psoas major muscle, and it enters underneath the inguinal ligament the vascular space to continue as the femoral artery.

Now we will focus on the upper part of the retroperitoneum and specifically on the visceral vessels of the abdominal aorta.

The first anterior branch of the abdominal aorta is the celiac trunk, located at the level of the twelfth thoracic vertebra, running above the body of the pancreas and the splenic vein.

It divides into the left gastric, the splenic and the common hepatic artery, that further divides into the proper hepatic and the gastroduodenal artery.

Now we move to the superior mesenteric artery, the second anterior branch of the abdominal aorta.

The superior mesenteric artery arises from the abdominal aorta immediately below the celiac trunk at the level of the first lumbar vertebra.

It runs dorsally to the body of the pancreas and the splenic vein. Then, it continues caudally, ventral to the uncinate process of the pancreas together with the superior mesenteric vein.

The superior mesenteric artery runs ventral to the 3rd part of the duodenum, enters the root of the small bowel mesentery and then divides in several terminal branches.

Please note that at its beginning, the superior mesenteric artery runs above the left renal vein.

We will now focus on the inferior mesenteric artery.

The inferior mesenteric artery is the last of the three major anterior branches of the abdominal aorta.

It originates at the level of the third lumbar vertebra below the caudal border of the duodenum, 3-4 cm above the aortic bifurcation.

The right and the left common iliac arteries arise from the abdominal aorta, ventral to the 4th lumbar vertebra.

The common iliac arteries are crossed ventrally by the superior hypogastric plexus and by the ureter at their terminal bifurcation.

The left common iliac artery is also crossed ventrally by the superior rectal artery, that is the terminal branch of the inferior mesenteric artery.

The external iliac artery originates from the common iliac artery at the level of the sacroiliac joint and it runs laterally along the medial border of the psoas major muscle.

Before passing underneath the inguinal ligament in the vascular space and becoming the femoral artery, the external iliac artery gives two branches: the deep circumflex iliac and the inferior epigastric arteries.

The external iliac artery may be crossed by the ureter, the ovarian vessels, the deep circumflex iliac vein and the round ligament.

The internal iliac artery is located dorsally to the ureter, ovary and fimbria.

 We are now approaching the iliac veins.

The femoral vein continues as the external iliac vein after its passage through the vascular space.

The external iliac vein joins the internal iliac vein to form the common iliac vein.

The confluence of the common iliac veins is located below the aortic bifurcation, at the level of the 5th lumbar vertebra.

 From a clinical point of view, it is important to know that the right ovarian vein drains directly to the inferior vena cava, while the left ovarian vein terminates into the left renal vein.

 Please note that the superior mesenteric vein, the inferior mesenteric vein and the splenic vein converge to form the portal vein behind the head of the pancreas.

**S2 - Femoral triangle**

The assessment of inguinal lymph nodes is based on the examination of the area called the femoral triangle, also called Scarpa’s triangle.

The medial side is the lateral border of the adductor longus muscle. The apex of the triangle corresponds caudally to the intersection of the adductor longus and sartorius muscle.

Its medial border forms the lateral side of the triangle, and the base of the triangle is the inguinal ligament.

The lateral space below the ligament is formed by the muscular space with the iliopsoas muscle and femoral nerve passing through it.

The medial space below the ligaments is formed by the vascular space with the femoral vein medially and the femoral artery laterally.

The floor of the triangle consists of the pectineus muscle medially and the iliopsoas muscle laterally.

We can further demonstrate medially the gracilis muscle and the adductor longus muscle, moving laterally we can describe sartorius muscle and outside the triangle: the vastus medialis muscle, the rectus femoris muscle, the vastus lateralis muscle, and the tensor fasciae latae muscle.

When assessing the femoral triangle apex on ultrasound, we should recognize dorsally the femoral vein and ventrally the femoral artery.

The femoral vein runs then medially to the femoral artery into the vascular space below the inguinal ligament.

When we continue underneath the inguinal ligament, we can follow the femoral vessels continuing as the external iliac vein and external iliac artery.

The branching of the inferior epigastric vein and inferior epigastric artery can be noticed.

 Going cranially, we see the junction with the deep circumflex iliac vein and artery.

Firstly, we will focus on the femoral vein. In the depth, it joins with the deep femoral vein, continuing together as femoral vein. More cranially and superficially it joins with the great saphenous vein at the saphenofemoral junction.

 At this level, a few centimeters under the inguinal ligament and after perforating the connective tissue of the cribriform fascia, the great saphenous vein drains to the femoral vein accompanied, with some anatomical variants, by the superficial external pudendal vein, the superficial circumflex iliac vein and the superficial epigastric vein.

Moving to the femoral artery, examining it at the distal end of the triangle, we notice how the artery moves from a ventral to a lateral position to the femoral vein, where it joins to the deep femoral artery, continuing as femoral artery in the vascular space.

It is important to know that the bifurcation of the femoral artery is approximately at the same level of the saphenofemoral junction, while the venous junction of the deep femoral vein and femoral vein is located distally.

 In the next step we assess lymph nodes starting from the superficial inguinal lymph nodes located above the cribriform fascia.

They form a vertical strand running along the great saphenous vein, including superficial epigastric vein and a horizontal lymphatic strand perpendicular at the level of saphenofemoral junction, running along the superficial circumflex iliac vein and superficial external pudendal vein.

 Now we will focus on the deep lymph nodes, which are located medially to the femoral vein, above the level of saphenofemoral junction and below the cribriform fascia, above the lower margin of the saphenous opening (also called fossa ovalis).

 Cranially the lymph nodes extend to the inguinal ligament where the uppermost node is called the Cloquet's node (or Rosenmuller).

**S3 - Superior diaphragmatic (cardiophrenic) lymph nodes**

Superior diaphragmatic, also called cardiophrenic lymph nodes, can be found in the fatty tissue of the lower part of the anterior mediastinum bordered by the base of the heart, the diaphragm, and the chest wall.

They are classified into three groups based on their location in relation to the heart.

We start the description from the anterior group.

The anterior group is located between the xiphoid process of the sternum, the costochondral articulation of the 7th rib and the pericardium.

The lateral group is located between the pericardium medially and the hilum of the lung where the phrenic nerve runs.

Now we will focus on the posterior group of the superior diaphragmatic lymph nodes.

The posterior group is located posteromedial to the Inferior vena cava next to the esophagus.

**S4 – Suplaclavicular lymph nodes and adjacent vascular anatomy**

As you can see in the scheme, the supraclavicular lymph nodes are shown on the left side of the neck, located in the supraclavicular fossa.

The supraclavicular lymph nodes can be found in the lateral cervical region, where the supraclavicular fossa is located.

The lateral cervical region is delimited by the posterior border of the sternocleidomastoid muscle anteriorly, the anterior border of the trapezius muscle posteriorly, by the superior border of the clavicle caudally.

The lateral cervical region is subdivided in two parts: a cranial part (the trigonum omotrapezium), and a caudal part (the supraclavicular fossa).

 The supraclavicular fossa, also called omoclavicular triangle, is delimited by the posterior border of the sternocleidomastoid muscle anteriorly, by the superior border of the clavicle caudally, and by the inferior belly of omohyoid muscle cranially, which separates it from the trigonum omotrapezium.

Here is underlined the omohyoid muscle.

 The lymph nodes can be found posterolateral to the internal jugular vein.

Focusing on the supraclavicular lymph nodes, highlighted here by the dotted circle, they are limited to the supraclavicular fossa.

 Now we move to the left side of the body, with the head positioned to your right.

The main structure in the lateral cervical region is the subclavian vein which joins the internal jugular vein forming the venous angle.

The thoracic duct opens in the left venous angle. It is also in this anatomical site that the supraclavicular Virchow’s lymph node is located.

 Other important structures in this region are the suprascapular artery and the transverse cervical artery.

 We can divide the subclavian artery in three parts according to its relation to the anterior scalene muscle: the first part is the interscalenic , medial to the scalene muscle, the second is the intrascalenic, dorsal to the scalene muscle and the third is the extrascalenic, lateral to the scalene muscle.

 Regularly, the suprascapular artery originates from the first part of the subclavian artery, and in this case the transverse cervical artery originates from the third part.

 Please note that the transverse cervical artery can also originate together with the suprascapular artery.

**S5 – Axillary and parasternal lymph nodes**

The brachial vein, along with the brachial artery, becomes the axillary vein at the humeral insertion of the teres major muscle, at the level of the caudal edge of the pectoralis major muscle which lies ventrally.

As it ascends, the axillary vein then becomes the subclavian vein at the level of the first rib laying ventral to the subclavian artery.

 On the right side of the armpit, we will introduce the borders of the axillary pyramid:

 The anterior wall is formed by the pectoralis major and pectoralis minor muscles (removed in the specimen), whose lateral edges define the anterior axillary fold.

The posterior wall is formed mainly by the scapula, subscapularis muscle and, in the lower part, the teres major and latissimus dorsi muscles, the latter define the posterior axillary fold.

 The medial border is delineated by the serratus anterior muscle and intercostal muscles.

 The lateral wall is formed by the humerus, coracobrachialis muscle and short head of the biceps muscle.

 The base of the axilla is formed by the skin, adipose tissue and axillary fascia.

The apex is defined by the first rib, clavicle and scapula.

The superior border of axilla corresponds to the inferior aspect of the clavicle and the first rib.

 Now we move to the left side of the body to demonstrate the axillary lymph nodes, classified into five groups and organized into three levels.

The axillary lymph nodes can be divided into three levels according to their relationship with the pectoralis minor muscle: the first level includes the lymph nodes lateral to the pectoralis minor muscle, the second level includes those underneath the muscle, and the third level includes those medial to it.

 In level one we recognize 3 groups of axillary lymph nodes:

The lateral, also called humeral, lymph-node group is found along the distal part of the axillary vein. The anterior group (also called pectoral) lymph-node group is found at the pyramid base, along the lateral thoracic vein. The posterior (also called subscapular) lymph-node group is located along the subscapular and thoracodorsal vessels.

 Lateral or humeral.

Anterior or pectoral.

Posterior or subscapular.

 Level two consists of the central lymph node group, at the center of the pyramid, along the middle part of the axillary artery.

 Finally, the apical lymph node group can be found at the level three, corresponding to the apex of the pyramid.

 First, we will focus on level one, starting with the lateral lymph nodes:

The lateral lymph node group is found along the distal part of the axillary vein.

To visualize it we can use as ultrasound landmarks the axillary artery and vein running deeply in the base of the pyramid.

The anterior or pectoral lymph-node group can be found behind the pectoralis major muscle and on the lateral aspect of the pectoralis minor muscle, running along and behind the lateral thoracic vessels.

 The ultrasound landmark for the identification of the anterior group is the lateral thoracic artery arising from the axillary artery and lateral thoracic vein.

 The posterior (or subscapular) lymph node group is located along the subscapular and thoracodorsal vessels. The lymph nodes are located in the posterior axillary fold on the tendon of latissimus dorsi and teres major muscle and on the subscapularis, muscle following subscapular vessels.

An important ultrasound landmark for this nodal group is the subscapular artery and vein which is joined by the thoracodorsal vein.

Now we move to level two, which consists of the central lymph node group.

These lymph nodes are located in the center of the pyramid, behind the pectoralis minor muscle, along the middle part of the axillary artery.

Finally, the apical group can be found at level three.

The lymph nodes of the apical group are located at the apex of the pyramid medially to the superior border of the pectoralis minor muscle, along the proximal part of the axillary vessels.

 In addition, at the center of the pyramid, the interpectoral lymph nodes also known as Rotter’s nodes can be found, in the layer between the pectoralis major and minor muscles.

Other important lymph nodes that may be infiltrated are the parasternal lymph nodes also called internal mammary (or internal thoracic) lymph nodes.

The parasternal lymph nodes are located anterolaterally to the internal thoracic vessels and are mainly found in the first five intercostal spaces.
